# A technique for in vitro fit assessment of multi-unit screw-retained implant restorations: Application of a triple-scan protocol

**DOI:** 10.1177/1758736012452181

**Published:** 2012-07-20

**Authors:** Stefan Holst, Matthias Karl, Manfred Wichmann, Ragai E Matta

**Affiliations:** Dental Clinic 2–Department of Prosthodontics, University Clinic Erlangen, Erlangen, Germany

**Keywords:** computer-aided design/computer-aided manufacturing, digital dentistry, fit assessment, implant, prosthetics

## Abstract

Recent advances in industrial non-contact scanners offer unprecedented opportunities for quality assessment of dental restorations. The majority of investigations published to date are limited to local two-dimensional results. A triple-scan protocol for virtual fit assessment of multi-unit screw-retained implant restorations is presented in this technical report. The advantages for application in biomechanical research include detailed three-dimensional information on internal component congruence in implant superstructures to be used in mathematical models.

## Introduction

It is generally accepted that absolute passive fit is unattainable for implant-retained multi-unit superstructures due to the required clinical and laboratory processing steps and manufacturing techniques.^1–4^ As clinical longevity and need for maintenance repair of implant restorations depend to a large extent on the precision of manufactured components, much research has focused on its improvement.^5,6^

Protocols such as external vertical gap measurements with microscopes, torque–angle signature analysis and strain gauge measurements are routinely used to assess precision of fit for implant frameworks.^7–9^ While these are well-established techniques, authors agree that the applied methodologies have shortcomings due to limited information display. One inherent shortcoming is the measurement of misfit in one or two linear dimensions, lacking the important third dimension.^10,11^

More recent studies have addressed the three-dimensional (3D) nature of the misfit.^9,10,12,13^ Non-contact optical precision scanners are used to digitize objects, and best-fit registration algorithms are used for virtual alignment. The underlying principle of these techniques, which are adopted from industrial quality control applications, is that the software algorithm attempts to achieve the greatest possible contact area of selected surfaces. However, dental products are free-form objects and distortion is always 3D. Thus, results may potentially be falsified, as areas of greater misfit are virtually approximated and may not accurately represent the actual discrepancy. The objective of the technique proposed here is to describe a registration protocol ensuring correct spatial orientation of components in relation to each other. The validity and reproducibility of this approach have been previously investigated and a similar protocol is applied for the virtual registration of conventional restorations for natural teeth.^11,14^

## Technique

**Figure 1.**
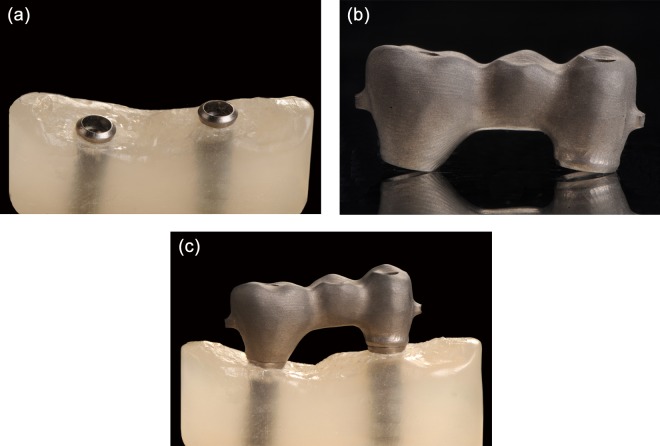
(a) Photograph of master cast containing the two implants, (b) implant framework and (c) screw-retained framework on master cast in actual position.

**Figure 2.**
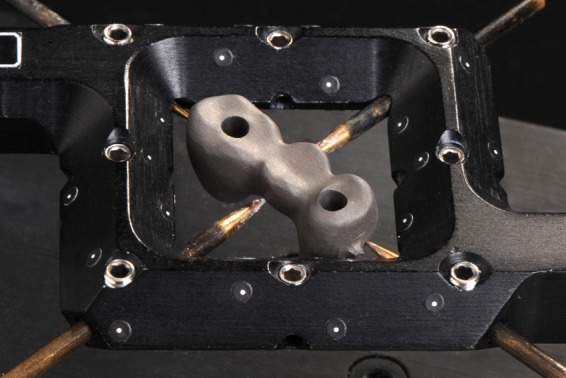
Photograph of top view of implant framework attached to the reference frame. The frame eliminates the need for markers on small objects and supports object digitization from both sides by integrating all measurements into a predefined coordinate system.

**Figure 3.**
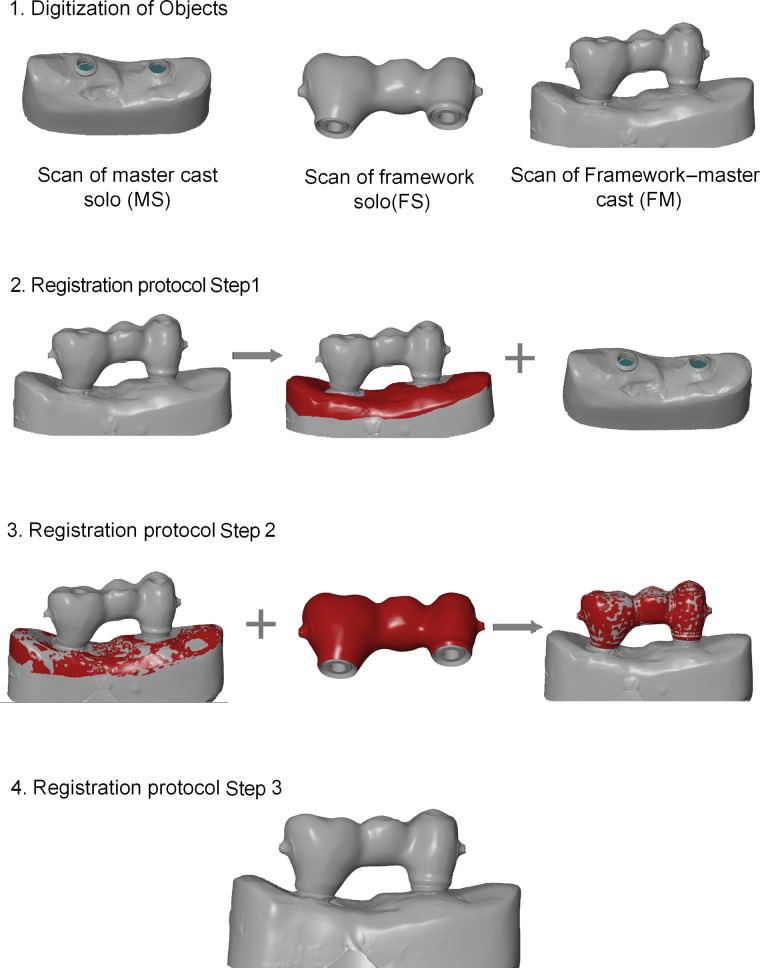
Display of the working process of the triple-scan protocol. Following digitization of objects, FM and MS are aligned in the first step. Note the red areas on master cast displaying the selected surfaces for subsequent best-fit registration. In the successive step, the previously selected areas are deselected (left image – Step 2) and surface areas are selected on FS, exclusively for registration with FM (Step 2). Screenshots of aligned virtual objects are shown. Note that the positioning represents true spatial orientation following the triple-scan protocol (Step 3). FM: framework–master cast; FS: framework solo; MS: master cast solo.

**Figure 4.**
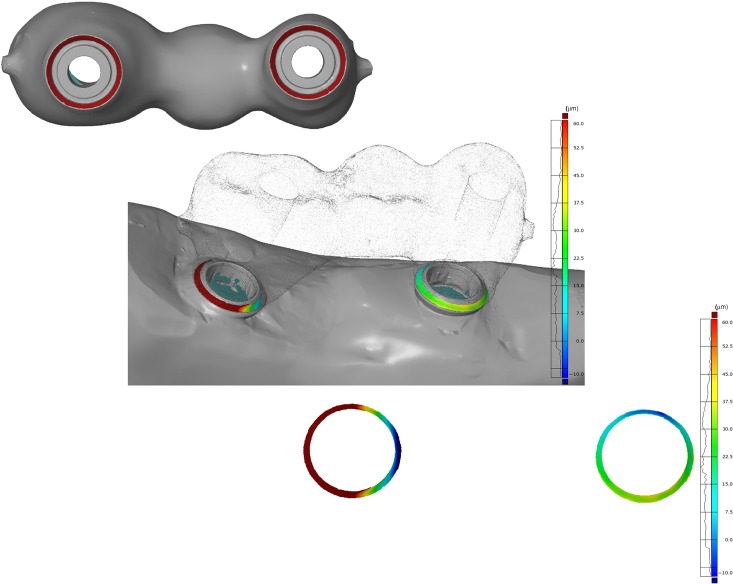
Screenshot of intaglio view of implant framework with exemplary surfaces selected for measurement of gap dimension (marked circles, top image). For illustration purposes, framework surface is made translucent, to display the location of measurement area (middle image) and exclusive view of measured gap dimensions with distribution chart (bottom image). Note the inhomogeneous distribution of gap dimensions.

1. Manufacture the implant master cast and the respective screw-retained test frameworks according to a defined research protocol (Figure 1(a) to (c)).
2. Modify the surfaces and eliminate implant replica reflectivity and frameworks under investigation to accommodate for non-contact optical digitization with either spray coating (e.g. titanium oxide) or surface roughening (e.g. sandblasting with 25 µm aluminium oxide powder, 1 bar pressure).
3. If surface coating is applied, utilize an airbrush system to ensure a homogenous and ultra thin coating layer.
4. Fully digitize components using an industrial high-precision non-contact scanner (e.g. ATOS II SO; GOM mbH, Braunschweig, Germany). For high-accuracy surface acquisition, choose the smallest measurement area available for the system.
5. Mount the frameworks under investigation in a special measurement frame (e.g. Reference frame; GOM mbH, Braunschweig, Germany) to allow for object digitization from both sides by integrating all measurements into a predefined coordinate system (Figure 2).
6. Attach scan markers (0.4 mm; GOM mbH, Braunschweig, Germany) to the implant master cast; scan the master cast solo (MS).
7. Scan and digitize the framework solo (FS).
8. Place the framework on the master cast, tighten framework abutment screws according to the research protocol and scan the framework–master cast (FM) object.
9. Use commercially available software (e.g. ATOS system software; GOM mbH, Geomagic Qualify, Research Triangle Park, NC, USA) to generate Standard Tessellation Language (STL) surfaces of the digitized objects (Figure 3).
10. Open the MS-STL and the FM-STL. Select the FM-STL as reference file and pre-align (pre-register) the data sets. For subsequent best-fit registration, select surfaces on the FM-STL, excluding the framework and the implant replicas and match data sets (Figure 3 – Step 1).
11. Apply the same protocol in a successive step, to match the FM-STL and the FS-STL. Select the FM-STL as reference CAD and pre-align (pre-register) data sets. For best-fit registration, select the entire outer contour of the FS, excluding the intaglio surfaces in contact with the implant replicas (Figure 3 – Step 2).
12. In the third and final step, delete the FM-STL and maintain the aligned FS-STL and MS-STL for subsequent virtual fit assessment according to the defined research protocol (Figure 3 – Step 3).
13. To measure gaps between contacting surfaces, select the intaglio surfaces of the framework in contact with implant surfaces or surfaces under investigation according to the research protocol and invert the surface normal (Figure 4).
14. With the implant replica of the master model defined as reference, measure distances to framework surfaces (Figure 4).

## Application of technique

### Experimental models

From a metal master model based on a patient situation with two implants (Standard Implant; Straumann, Waldenburg, Switzerland), 20 polyether impressions (Impregum; 3M ESPE, St Paul, MN, USA) were taken with custom trays. Following established protocols for manufacture of definitive gypsum master casts, models were randomly divided into two groups (*n* = 10/group). Group I conventional cast-on abutments (synOcta Goldsekundärteil; Straumann, Waldenburg, Switzerland) were used for manufacture of three-unit screw-retained high-noble alloy frameworks. The frameworks were manually adapted on the respective gypsum model, and premature contacts resulting from the casting process were eliminated.

In group II, implant replicas were digitized utilizing a contact-probe scanner (Carl Zeiss, Wetzlar, Germany) and screw-retained titanium frameworks were CAD/computer-aided manufacturing (CAM) produced (NobelProcera Implant Bridge Titanium; Nobel Biocare, Zurich, Switzerland). Following the previously described triple-scan digitization and registration protocol with an industrial non-contact scanner (ATOS SO II), superstructure fit was analysed three dimensionally on each respective master cast. For high-accuracy surface acquisition, the smallest available measurement area was chosen (30 × 24 mm^2^) and frameworks were mounted in special measurement frames (Reference frame). All tests were conducted at an ambient room temperature of 20°C.

### Data matching and analysis

**Figure 5.**
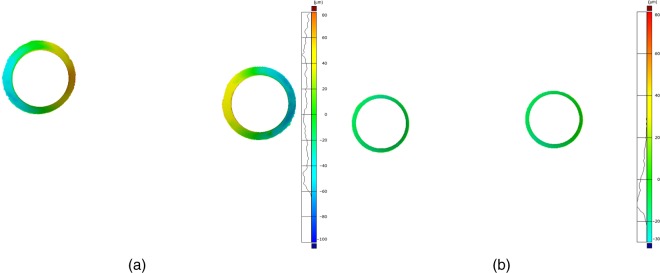
Exemplary screenshot of ROIs. (a) The deformation of the cast superstructure with an increased gap size towards the pontic area is clearly visible. (b) The CAD-/CAM-manufactured superstructure reveals equal circumferential fit. ROIs: regions of interest; CAD/CAM: computer-aided design/computer-aided manufacturing.

For fit assessment, only the contacting surfaces of the implants and the framework were selected. By inversion of the normal of the framework contact area and selection of the corresponding implant surface area as reference, the distances between surfaces were measured (Figure 5(a) and (b)). To eliminate an estimated measurement error – digitizing system and the spray coating – all values ≤10 µm were regarded as full contact between mating components. In a second analytical step, the percentage of contacting area (in square millimetre) ≤10 µm was calculated for each model.

For statistical analysis, implants were virtually sectioned in half (bucco-oral direction) to differentiate between surface areas towards the pontic site and surface area towards the non-pontic sites and resulting mean distances (millimetre) calculated. Statistical methods applied included the two-sided and exact Mann–Whitney *U* test for between-method comparisons and exact Wilcoxon signed-rank test for outer–inner area comparisons; *p* values of *p* ≤ 0.05 refer to an exploratory significant difference.^15^

## Results

**Figure 6.**
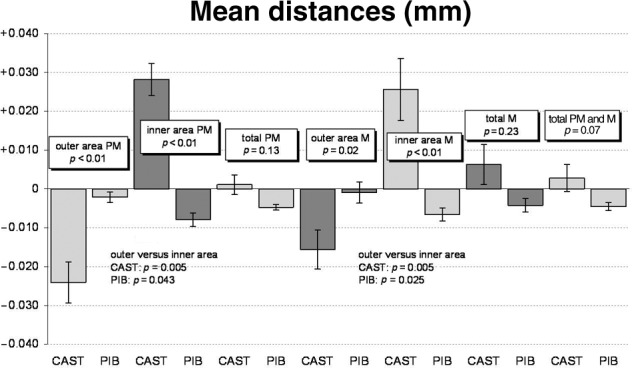
Descriptive statistics of mean differences between various ROIs in both cast superstructure (CAST) and CAD-/CAM-manufactured restoration (PIB).

CAM-machined restorations exhibited significantly better fit than cast restorations (*p*<0.001). Fit assessment of contacting surfaces for group I indicated shrinkage towards the pontic site, whereas for group II equal circumferential fit was observed (Table 1 and Figure 6). These differences between manufacturing techniques were not found when the total surface areas were analysed.

**Table 1.** 

Mean distances (mm)	Group	Mean	Standard error	Minimum	Median	Maximum	Cases	*p* Value
Outer area PM	CAST	−0.024	0.005	−0.047	−0.021	0.003	10	*p* < 0.01
	PIB	−0.002	0.001	−0.008	−0.003	0.006	10	
Inner area PM	CAST	0.028	0.004	0.010	0.030	0.046	10	*p* < 0.01
	PIB	−0.008	0.002	−0.019	−0.008	0.000	10	
Total PM	CAST	0.001	0.003	−0.010	0.000	0.014	10	*p* = 0.13
	PIB	−0.005	0.001	−0.007	−0.005	0.000	10	
Outer area M	CAST	−0.016	0.005	−0.046	−0.013	0.006	10	*p* = 0.02
	PIB	−0.001	0.003	−0.015	−0.001	0.012	10	
Inner area M	CAST	0.026	0.008	−0.005	0.024	0.082	10	*p* < 0.01
	PIB	−0.007	0.002	−0.018	−0.006	0.000	10	
Total M	CAST	0.006	0.005	−0.013	0.005	0.045	10	*p* = 0.23
	PIB	−0.004	0.002	−0.016	−0.003	0.003	10	
Total PM and M	CAST	0.003	0.003	−0.010	−0.002	0.026	10	*p* = 0.07
	PIB	−0.005	0.001	−0.011	−0.004	0.000	10	

CAD: computer-aided design; CAM: computer-aided manufacturing; inner area = towards the pontic; outer area: mesial/distal areas of the bridge; M: molar; PIB: procera implant bridge; PM: premolar; ROIs: regions of interest; total: average of inner and outer areas. The PM and M implant surfaces were virtually cut into half resulting in four different areas for fit assessment.

The fit assessment (mean distances between implants and superstructure) clearly shows the three-dimensional distortion of the cast superstructures (CAST) towards the pontic area in both the PM and M areas of the restoration, while the CAD-/CAM-manufactured superstructure (PIB) revealed equal circumferential fit.

CAD/CAM: computer-aided design/computer-aided manufacturing; PM: premolar; M: molar; PIB: procera implant bridge.

Description of areas investigated: inner area = towards the pontic; outer area = mesial and distal areas of the bridge; total = average inner and outer areas.

## Discussion

The presented triple-scan and registration technique may be applied for future in vitro research projects, because the methodology eliminates a number of shortcomings inherent to currently applied investigative techniques.^7–10^ It is non-destructive and allows for repeated measurement of the same object (e.g. prior to and after veneering or variation in screw tightening sequences). Currently, this is usually done with two-dimensional external vertical gap measurement techniques. However, microscopic analysis is limited to the outermost area of components and does not yield information on internal component fit of mating surfaces.^7^ By utilizing the external surfaces for data matching of the three scans, the transformation error during registration can be greatly reduced and established registration algorithms applied.^14^ This differs from other reported approaches for 3D fit assessment of components that require additional materials and highly complex alignment and registration procedures.^9,10,13^

This approach can be easily applied in a routine laboratory setting without the need for specific metrology expertise because no complex alignment and registration procedures are needed and established best-fit registration algorithms can be applied.^11,14^ A notable advantage compared to other reported techniques in dental research is that the true spatial orientation of a framework attached to the respective definitive cast is ensured. The danger of eliminating singular irregularities through a general best-fit protocol is eliminated. While the application range of 3D-assessment techniques in dentistry is broad, certain system-intrinsic and object-related factors must be taken into account when protocols are applied. If highly reflective or translucent materials are used, the surface may need to be coated, possibly introducing an additional source of measurement error secondary to system-related measurement accuracy. However, if comparative studies are performed, the absolute values are of minor importance. If high-accuracy measurements are required, high-precision contact-probe scanners could be used for verification purposes.^16^ Other options include scan systems with a working principle that is independent of material or surface quality or special surface-coating appliances that can deposit defined and homogeneous surface layers. Further studies are needed to investigate the intrinsic measurement system and the protocol-related effects on the measurement accuracy.

The pilot investigation applying the described technique showed that the applied triple-scan protocol and the 3D precision assessment is a valid protocol to obtain detailed information on true fit of components at selective regions of interest. Based on the initial results, conclusions of other scientific publications indicating the superior precision of CAD-/CAM-manufactured components compared to conventional casting techniques for implant-supported/implant-retained superstructures can be confirmed.^1,4,17^ It is important to note that the differences between manufacturing techniques were not statistically significant if overall (total area) fit was assessed but only if surfaces were investigated separately. This finding underlines the importance of more detailed and sophisticated analytical methods, as an average of 3D distorted components does not necessarily reflect the true congruence/incongruence between two components.

## Summary

The presented technique is an easy-to-use method facilitating the 3D internal fit and precision measurements of implant restorations. It can be used to provide valuable data on manufacturing distortion and misfit of implant-retained restorations.
